# Merkel cell carcinoma: a forty-year experience at the Peter MacCallum Cancer Centre

**DOI:** 10.1186/s12885-022-10349-1

**Published:** 2023-01-07

**Authors:** Annie J. Wang, Brendan McCann, William C. L. Soon, Paolo B. De Ieso, Mathias Bressel, Andrew Hui, Margaret Chua, David L. Kok

**Affiliations:** 1 Department of Radiation Oncology, Peter MacCallum Cancer Centre, 305 Grattan St, Melbourne, Victoria 3000 Australia; 2 Icon Cancer Centre Moreland John Fawkner Private Hospital, Coburg, Australia; 3 Centre for Biostatistics and Clinical Trials, Peter MacCallum Cancer Centre, Melbourne, Victoria Australia; 4 Andrew Love Cancer Centre, Geelong, Victoria Australia; 5 Department of Clinical Pathology, The University of Melbourne, Melbourne, Victoria Australia

**Keywords:** Merkel Cell Carcinoma, Management, Surgery, Radiotherapy, Immunotherapy

## Abstract

**Background:**

Merkel cell carcinoma (MCC) is a rare but highly aggressive neuroendocrine skin malignancy, with Australia having the highest reported incidence in the world. There is currently a lack of consensus regarding optimal management of this disease.

**Methods:**

This was a retrospective audit conducted by reviewing existing medical records of MCC patients presenting to the Peter MacCallum Cancer Centre (PMCC) between 1980 and 2018. The primary endpoint was locoregional recurrence. The secondary endpoints were distant recurrence, disease-free survival (DFS) and overall survival (OS).

**Results:**

A total of 533 patients were identified. Locoregional recurrence occurring at one, two and 5 years was 24, 31 and 32%, respectively. The estimated 5-year OS and DFS were 46% (95% Confidence Interval [CI] 41–51%) and 34% (95% CI 30–39%) respectively. Older age at diagnosis (hazard ratio [HR] per year = 1.07, 95% CI 1.06–1.07, *p* < 0.001), and larger primary tumour diameter (HR =1.16, 95% CI 1.03–1.31, *p* = 0.019) were associated with worse OS on multivariable analysis. Positive or negative histopathological margin status was not associated with OS or DFS differences in patients treated with post-operative radiotherapy.

**Conclusions:**

In our study, about a third of patients developed locoregional recurrence, distal recurrence or both, and there appears to be no change over the last four decades. If treated with adjuvant radiotherapy, there is no difference in OS or DFS with positive surgical margins. Findings should influence future guidelines.

**Supplementary Information:**

The online version contains supplementary material available at 10.1186/s12885-022-10349-1.

## Background

Merkel cell carcinoma (MCC) is an uncommon but highly aggressive cutaneous neuroendocrine tumour.

Advanced age, immunosuppression, Merkel cell polyomavirus and ultraviolet light (UV) exposure are risk factors [1–3] and Australia has the highest incidence in the world [4] due to the higher UV index compared with other countries [1, 5, 6]. The mortality rate of patients with MCC is significantly higher than that seen in melanoma [7] and global incidence increasing.

While there is a growing body of evidence in MCC, this is mostly constrained to relatively low level evidence and thus, ‘optimal management’ of MCC still remains the subject of some debate. For stages I-III, surgery with a 1-2 cm margin is the recommended primary treatment according to international guidelines, with radiotherapy (RT) reserved for post-operative high-risk cases, close margins or inoperable patients [8, 9]. MCC has also been shown to be uniquely radiosensitive [10], which has led to an established role of radiation therapy in primary management particularly in Australia and New Zealand [11]. Additionally, while the role of cytotoxic chemotherapy is looking increasingly limited in MCC management, immunotherapy appears promising, with a proven role in metastatic disease [12] and with data from Stage I-III adjuvant trials currently awaited [11, 13].

The Peter MacCallum Cancer Centre (PMCC) in Melbourne was the first quaternary referral cancer centre established in Australia. Over the past 40 years it has cared for the largest documented MCC patient population in the Southern Hemisphere and maintained records of their diagnosis, management and clinical outcomes. As such, the institute is well positioned to detail and analyse the outcomes of patients with MCC and help contribute to the international knowledge base regarding MCCs diagnosis and management. Here, we present findings of our forty-year experience with a particular focus on the loco-regional control and survival outcomes of patients, and the influencing management factors.

## Methods

This was a retrospective case series conducted by reviewing existing electronic medical records of MCC patients presenting between 1980 and 2018. We evaluated diagnostic work up, treatment received and outcomes as part of routine care. This study updates a previous analysis of 176 patients with MCC treated at PMCC between 1980 and 2006 that was published in 2011 [14]. All patient data retrieved from the PMCC electronic medical records system was recorded into a centralised database. Inclusion into the study required a histopathological diagnosis of MCC and treatment at PMCC with a minimum follow up time of 12 months. Patients who presented with recurrent locoregional disease after previous biopsy or surgery outside our centre were also included. All patients were re-staged in accordance with the American Joint Committee on Cancer (AJCC) 8th edition staging system (2017). The study was conducted according to the NHMRC National Statement on Ethical Conduct in Human Research (2007 and updates) and the World Medical Association Declaration of Helsinki (2013 and updates). Institutional ethics approval was sought prior to any data collection (Approval number and granted on the 22nd of October 2018 in accordance to PMCC research governance requirements - Project Number 18/225R). Informed consent was waivered due to the retrospective nature of the study, this was approved by the PMCC ethics committee.

The primary endpoint was locoregional recurrence, defined as a recurrence that occurred at either the local (primary skin site) or regional (draining lymphatic system) site. Patients without locoregional recurrence were censored at the last date of disease assessment and death was considered a competing event when calculating the cumulative incidence. The secondary endpoints were distant recurrence, disease-free survival (DFS) and overall survival (OS). Histopathological margins were as per pathology report.

Statistical analyses were performed in R 3.6.0 [15] using standard and validated statistical procedures. The Kaplan-Meier method was used to estimate OS and DFS. Estimates and associated 95% confidence intervals at key time points were reported. Local, loco-regional and distant recurrences were described as cumulative incidences with death as a competing risk. Cox proportional hazard models were used to assess the impact of possible prognostic factors on OS and DFS.

## Results

### Patient Population Characteristics

533 patients (315 male, 218 female) treated for MCC at PMCC between 1980 and 2018 were included in this study. Median follow-up for patients was 5.3 years. The distribution of patient characteristics is as listed in Table 1. The median age at diagnosis was 78 years (range, 19–98). Most primary tumours were located on the head and neck (50%) and 69/533 patients (13%) presented with regional nodal disease without an identified primary tumour. The median primary tumour size was 15.0 mm (range, 1.0–180.0 mm).

**Table 1:** Clinicopathological characteristics

Characteristic, ***N =*** 533	n (%), range
**Age at diagnosis, yrs**	(yrs)
Mean (sd)	76.1 (11.9)
Median [range]	78 [19–98]
Interquartile range	69–85
**Sex**	
Male	315 (59)
Female	218 (41)
**Immunosuppressed**	
Yes	77 (14)
No	456 (86)
**Other Skin Malignancy**	
Yes	272 (77)
No	81 (23)
Missing	180
**Sun damaged skin**	
Yes	112 (93)
No	9 (7)
Missing	412
**Viral Status**	
Positive	38 (39)
Negative	60 (61)
Missing	435
**Location of Primary**	
Head & Neck	267 (50)
Upper Limbs	77 (14)
Lower Limbs	95 (18)
Trunk	24 (5)
Unknown	70 (13)
**Unknown primary site**	
Yes	69 (13)
No	464 (87)
**Median Tumour diameter, mm, (IQR)**	15 (9–23)
**Stage (AJCC 8th edition)**	
I	225 (43)
II	72 (14)
III (A or B)	215 (21)
IV	12 (2)
Missing	9
**Margins**	
Negative	288 (67)
Positive	145 (33)
Missing	100
**Staging modality**	
Chest X-ray	47 (10)
CT	286 (60)
PET	300 (58)
**Treatment received**	
Surgical excision alone	34 (6)
Surgery + RT	393 (74)
RT/CRT	79 (15)
Other	27 (5)

Merkel Polyomavirus testing was performed in 98 patients, of which 39/98 patients (40%) were virus positive. A history of synchronous or previous cutaneous skin malignancy was reported in 272/353 (77%) documented cases and 77/533 patients (14%) were recorded to be immunosuppressed. This was mostly due to concurrent immunosuppressant medication (54 patients) with a further 18 patients having a haematological cause and the remaining due to other comorbidities.

### Staging

524 patients had some or all of their staging at PMCC. PET scanning has become more prevalent for diagnostic work up over the last 10 years. Between 2010 and 2019, 196/251 patients (78%) received PET; an absolute increase by 25% compared to the previous decade. N1b stage (image-detected lymphadenopathy) also increased during that time (16% vs 26%). See Supplementary online data – Table e1 for more information.

A majority (43%) had Stage I disease (no more than 2 cm across and not spread to lymph nodes). Only 12 patients (2%) presented with distant metastatic disease as this was routinely managed at their local centre.

### Sentinel lymph node biopsy

Sentinel lymph node biopsy (SLNB) was performed in 68 patients at the PMCC of which there was pathological information available for 66. For patients with T1 disease, 60 SLNB were performed which returned 16/60 positive (27%) and 42/60 negative (70%) for disease with sentinel node status unknown for 2 cases. For T2 patients, 8 SLNB were performed, and of those 3/8 were positive (38%) and 5/8 were negative (63%). See Supplementary online data – Table e1 for more information.

### Surgery

As first modality, surgery with therapeutic excision was performed on 454/533 patients (85%) with histopathological margin status known for 433 patients. Median pathological margin was 2.0 mm [0.0–40.0 mm] with positive pathological margins reported in 145/433 patients (33%), 154 patients underwent nodal surgery, of these 70/154 (45%) were biopsies (not including SLNB which were considered separately), 56/154 were nodal level dissections (36%) and 28/154 were local node excisions (18%). Overall, only 34/533 patients (6%) were treated with surgery as a single modality of which 26/34 (76%) were stage I or II.

### Radiotherapy

66/533 patients (12%) were initially treated with definitive RT (9%) or combined chemo- RT (3%) with carboplatin and etoposide. 16 patients had Stage I disease, 12 had Stage II disease, 36 had Stage III disease and 2 had Stage IV disease. Following excision, 383/454 patients (84%) received post-operative RT to the primary and/or nodal regions.

Table 2 lists collated radiotherapy treatment details for patients who received radiotherapy as part of their treatment course. For patients who received adjuvant RT and definitive RT the median dose and number of fractions delivered was 50Gy in 25 fractions. For patients who received definitive radiotherapy the median dose was 54Gy and median number of fractions was 27. See discussion for a review of our indications for radiotherapy.

**Table 2:** Radiotherapy treatment details

Treatment Group	Local excision - > RT (***n =*** 291)	WLE - > RT (***n =*** 92)	Definitive RT (***n =*** 47)	Palliative RT (***n =*** 13)
**RT dose to primary/met/intransits Site (Gy)**				
Median [range]	50.0 [2.5–60.0]	50.0 [8.0–60.0]	50.0 [8.0–60.0]	30.0 [21.0–30.0]
IQR	40.5–50.0	45.0–50.0	45.0–55.0	24.0–30.0
Missing	28	4	12	4
**RT fractions to primary/met/intransits Site**				
Median [range]	25.0 [1.0–31.0]	25.0 [1.0–30.0]	25.0 [3.0–30.0]	5.0 [3.0–10.0]
IQR	15.0–25.0	15.0–25.0	19.0–27.5	5.0–9.0
Missing	28	4	12	4
**RT dose to node (Gy)**				
Median [range]	50.0 [2.5–63.0]	50.0 [20.0–68.0]	54.0 [36.0–60.0]	30.0 [20.0–40.0]
IQR	45.0–50.0	46.0–50.0	50.0–60.0	29.2–30.0
Missing	83	24	14	5
**RT fractions to node**				
Median [range]	25.0 [1.0–31.0]	25.0 [5.0–33.0]	27.0 [12.0–30.0]	7.0 [5.0–16.0]
IQR	15.8–25.0	20.0–25.0	25.0–30.0	5.0–10.0
Missing	83	24	14	5

*RT* Radiotherapy, *WLE* Wide Local Excision, *IQR* Interquartile Range

### Immunotherapy

Of the 533 patients identified, 26/533 (5%) received immunotherapy during their treatment course for recurrence.

### Recurrence and Survival

The cumulative incidence of local recurrence at one, two and 5 years were 8, 10 and 10% respectively. Locoregional recurrence occurring at one, two and 5 years was 24, 31 and 32%, respectively. Distant recurrence occurring at one, two and 5 years was 16, 25 and 30% respectively. See supplementary online data – Table e2 for more information.

**Fig. 1:**
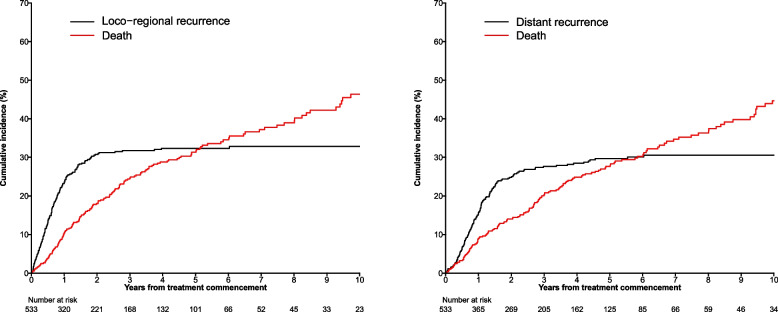
Cumulative Incidence of Loco-regional Recurrence, Distant Recurrence and Death

Figure 1 demonstrates the relationship between locoregional and distal recurrence and death. The estimated five-year OS was 46% (95% CI = 41–51%). The estimated five-year DFS was 34% (95% CI = 30–39%).

292/533 patients (55%) died during follow up. There was information on cause of death available in 161/292 deaths of which 106/161 (66%) were attributed to MCC and 55/161 (34%) were from other causes.

**Fig. 2:**
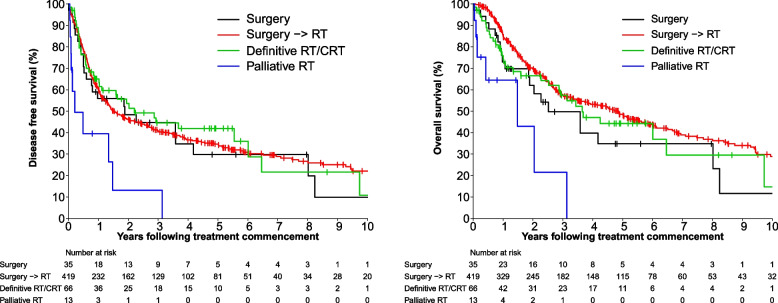
Disease Free Survival and Overall Survival by initial treatment modality

Difference in DFS and OS by modality of treatment is outlined in Fig. 2. The one and 5 year overall DFS estimates with surgery alone were 56% (95% CI =38–71%) and 30% (95% CI =14–48%) respectively. Those who were treated with definitive RT or chemo-RT had one and five-year DFS of 65% (95% CI =52–75%) and 42% (95% CI =28–55%). Patients who were treated with surgery followed by adjuvant RT had one and five- year DFS of 60% (95% CI =55–65%) and 35% (95% CI 30–40%).

The one and five- year OS estimates with 95% confidence intervals with surgery alone were 73% (95% CI = 54–85%) and 35% (95% CI = 17–54%). Definitive RT or chemo-RT had one- and five-year OS of 76% (95% CI = 63–85%) and 44% (95% CI = 30–58%). Patients who were treated with surgery followed by adjuvant RT had one and five- year OS of 85% (95% CI = 81–88%) and 48% (95% CI = 43–54%).

Those with MCC of unknown primary had improved DFS but not OS compared to a known cutaneous MCC primary (see supplementary online data Figure e1).

**Fig. 3:**
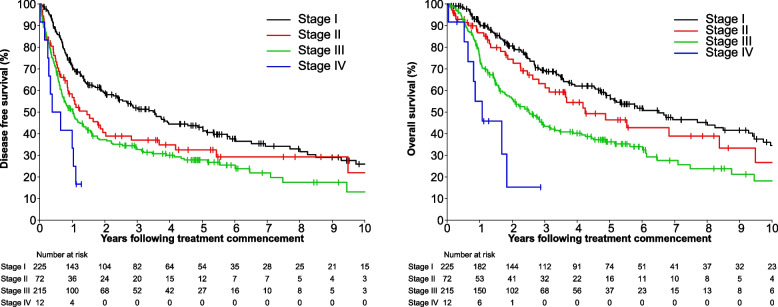
Disease Free Survival and Overall Survival by stage

Table 3 demonstrates univariable and multivariable analysis on the prognostic variables of age, sex, immunosuppression, tumour size, histopathological margins and nodal status. Positive histopathological margins were not significant for DFS (HR = 0.84, 95% CI: 0.64–1.11, *p* = 0.223) or OS (HR = 0.94, 95% CI: 0.70–1.28, *p* = 0.706). Increasing age and immunosuppression both demonstrated an association with OS and DFS. Tumour size predicted for OS (HR = 1.16, 95% CI 1.03–1.31, *p* = 0.019) but not DFS (HR 1.07, 95% CI 0.96–1.19, *p* = 0.235) and nodal status being the most significant adverse factor for DFS (HR = 2.36, 95% CI: 1.80–3.10, *p* = < 0.001) and OS (HR = 2.28, 95% CI: 1.68–3.09, *p* = < 0.001). Figure 3 also demonstrates that earlier staging results in improved outcomes however over the last four decades, we have not observed any difference in DFS or OS (see supplementary online data Figure e2, Table e3–5).

**Table 3:** Prognostic variables associated with overall survival and disease free survival

			Overall Survival				Disease Free Survival			
			Univariable		Multivariable		Univariable		Multivariable	
**Variable**	**Level**	**N**	**HR (95% CI)**	***p*-value**	**HR (95% CI)**	***p-value***	**HR (95% CI)**	***p-value***	**HR (95% CI)**	***p-value***
Age, years	Per unit increase	533	1.06 (1.05, 1.08)	< 0.001	1.07 (1.06, 1.09)	< 0.001	1.04 (1.03, 1.05)	< 0.001	1.05 (1.03, 1.06)	< 0.001
Sex	Female	218	ref	0.019	ref	0.123	ref	0.204	ref	0.598
	Male	315	1.33 (1.05, 1.68)		1.27 (0.94, 1.71)		1.15 (0.93, 1.42)		1.07 (0.83, 1.40)	
Immunosuppression	No	456	ref	< 0.001	ref	< 0.001	ref	0.041	ref	0.043
	Yes	77	1.76 (1.29, 2.41)		2.59 (1.73, 3.89)		1.34 (1.01, 1.79)		1.49 (1.03, 2.16)	
Tumour size, cm	Per unit increase	401	1.11 (1.04, 1.18)	0.002	1.16 (1.03, 1.31)	0.019	1.06 (0.99, 1.13)	0.089	1.07 (0.96, 1.19)	0.235
Margins	Negative	288	ref	0.338	ref	0.706	ref	0.801	ref	0.223
	Positive	145	1.14 (0.87, 1.49)		0.94 (0.70, 1.28)		0.97 (0.76, 1.24)		0.84 (0.64, 1.11)	
Nodal status	Negative	302	ref	< 0.001	ref	< 0.001	ref	< 0.001	ref	< 0.001
	Positive	231	1.75 (1.39, 2.21)		2.28 (1.68, 3.09)		1.64 (1.33, 2.02)		2.36 (1.80, 3.10)

## Discussion

To our knowledge, this is the largest study to date of patients with MCC treated at a single institution. In 2011, we reported on 176 patients with MCC with a median follow-up of 24 months [14] our current analysis expands on this by including 533 patients over four decades with a median follow up of 64 months.

Being a single institution case series has advantages that comes from the relative uniformity of record keeping at PMCC as well as a having a highly protocol driven approach to the diagnosis and management of this disease. Our internal PMCC unit treatment policies have been consistent with the Australia/New Zealand wide trial protocols TROG 96.07 [13] and TROG 09.03 [16] which we were heavily involved in, and the largest recruiter to both these studies.

Our treatment protocol advocates surgery to obtain a histological diagnosis, but no requirement to obtain clear margins. Patients that are T1N0 with ≥2 cm clear margins and a negative SLNB and PET require no adjuvant radiotherapy. T1N0 with < 2 cm margins and a negative SLNB and PET receive adjuvant radiotherapy to the primary site. All ≥T2 patients that are N0 receive radiation to the primary site and the first draining nodal echelon. All N+ patients receive radiation the involved nodal region and the first draining nodal echelon beyond that. This protocol was documented and published as part of TROG 09.03 [16]. Our radiation dose for macroscopic disease has increased over the decades, from 50Gy in 25 fractions in the 1990s, to 54Gy in 27 fractions (usually with chemotherapy as part of TRIOG 09.03) from 2009 and 60Gy in 30 fractions from 2015. Our radiation dose for microscopic disease has remained at 50Gy in 25 fractions throughout this time. As the majority of our patients (454/533) received an excision of the primary prior to radiotherapy it is unsurprising that are most frequent radiation prescription dose was 50gy in 25 fractions.

Our findings demonstrate similarities to other single institution findings [17–20] in that MCC is a disease of the elderly, occurs most commonly in the head and neck region and prognosis is negatively influence by increasing tumour size and stage at presentation. Other consistencies include the high rate of recurrence, both locoregional and distal. We also corroborated the use of sentinel lymph node biopsy in upstaging patients and found that increasing use of PET scanning found more radiologically diagnosed nodal disease.

The sentinel lymph node biopsy (SLNB) rate in our patient cohort was 66/533 patients. There were three different factors which contributed to this low rate of SLNBs in our patient population. Firstly, PMCC is a quaternary referral center and a significant numbers of patients have been operated on by external surgeons and reconstructed prior to referral. Due to the uncertainties of performing SLNB post-reconstruction we do not routinely perform them in this circumstance if they have clear PET imaging. Secondly, as described above, it is our protocol to treat all patient with ≥T2 tumours with elective nodal irradiation due to the high risk of nodal recurrence, hence SLNB are only performed on T1N0 patients. Finally, the SLNB paradigm was introduced to the service in the mid-2000s in line with the international literature at the time. Hence, patients treated between prior to the year 2000 were not routinely biopsied (see Table e1 for breakdown by decade). Our approach to SLNB since then is protocolised and all relevant data is in fact being prospectively collected in the internal prospective phase II SLNB trial ‘BiopsyME’ (PMCC15–57).

This study presents useful data on treatment outcomes that may assist the refinement of international best-practice guidelines. In particular it raises questions over the optimal surgical approach in early stage (I-II) MCC. A recent analysis on the National Cancer Database suggests that surgical margins larger than 1 cm are associated with better overall survival [8, 21] and re-excision is recommended in positive margin disease [9, 22]. Our study here does not include clinical surgical margins but has significant robust data on histopathological margins in the setting of post-operative RT. We found no difference in overall or disease free survival between patients who had positive or negative histopathological margins and received post-operative RT. This is in keeping with the Seattle group’s work based on MCC specific survival [23]. Our data poses the question, should 1-2 cm surgical margins still be recommended? Especially with additional evidence that RT improves locoregional control and overall survival [24] and that skin graft of flaps can delay commencement of RT [25]. Therefore, a more streamlined approach advocating only minimally invasive, primary closure surgery, without an emphasis on pursuing clear margins may be all that is necessary in patients planned for post-operative RT.

Some centres have already taken this approach and recommend that in MCC with high risk factors (primary tumours > 1 cm diameter, LVI, positive SLNB, chronic immunosuppression such as lymphoma/leukaemia, head and neck tumours) patients should not have re-excision in the setting of a close or positive margin if planned for adjuvant RT [11, 26]. In many Australian centres, including PMCC, the practice has gone one-step further and often RT is the primary modality of treatment for stage I-III MCC after histological diagnosis [11]. Ultimately, higher level evidence will be needed to definitively answer these questions on what is the optimal management pathway for patients with MCC.

Given the uncertainties which still exist in MCC management, multidisciplinary discussion, including input from a specialised surgical oncologist, radiation oncologist and medical oncologist, supported by medical imaging and pathology is vital for these patients. The recurrence rate and overall survival from MCC remains relatively poor compared to other skin cancers, albeit in an elderly population with co-morbid disease. In our study, about a third of patients developed locoregional recurrence, distal recurrence or both, and there appears to be no change over the last four decades. Immunotherapy has shown to be a paradigm shift in the treatment of metastatic MCC [27, 28] and is currently being trialled as adjuvant treatment in the I-MAT study (NCT04291885) in Australia and the STAMP (NCT03712605) and ADAM (NCT03271372) trial in America. In our study here, only a small number (5%) of patients received immunotherapy and therefore our data could be useful as a pre-immunotherapy era ‘snapshot’ of outcomes.

There are a number of limitations worth noting in this study. Being a single institution study is both a strength and weakness of this study. Despite the (previously described) advantages of uniform record keeping, it also introduces an inherent bias that come from a lack of external validation. In addition, it is retrospective and therefore dependent on quality of the medical records kept. As expected this resulted in some inconsistencies seen in the reporting of pathology from external pathology providers, incomplete follow-up, missing diagnostic and treatment information. In addition, as there was heterogeneity amongst certain variables such as RT dose, tumour size and margin size it was not feasible to analyse these in depth in this current study.

The 40 year time period covered by this study was a strength of this study, but also introduced some inherent issues relating to the staging of disease. 7 different AJCC staging systems were in use throughout this time, reflective of both evolving diagnostic practices and clinical understanding of the disease. For ease of reference and continuity of analysis we have classified all patients using the current AJCC 8th edition staging system but note that this comes with the potential risk of stage migration [29]. Stage migration raises the possibility of underestimating clinical outcomes of each staging group which should be considered when reviewing the stage-by-stage outcome data as presented in Fig. 3. In addition, improved treatment techniques over the 40 year period mean that the amalgam data presented here is likely an underestimation of the clinical outcomes of patients treated in the modern era, but still serves as an important baseline and representative picture of the clinical courses of these patients to date.

## Conclusion

Our study described the staging, treatment and patient outcomes of over 500 MCC patients managed at the Peter MacCallum Cancer Centre. Of particular interest was the finding that surgical pathological margin status did not impact on clinical outcome in the setting of high utilisation of post-operative RT. This suggests that pursuit of clear margins may not be necessary, particularly where it is technically or clinically difficult to achieve. This data will aid in guiding future trials, clinical practice, and the refinement of clinical guidelines for this uncommon disease.

### Supplementary Information

**Additional file 1.**

## Data Availability

Data supporting the results can be found in the supplementary material. Please contact author Dr. David Kok (David.kok@petermac.org) if you wish to request data from this study.
