# Supplementary material for: Merkel cell carcinoma: a forty-year experience at the Peter MacCallum Cancer Centre
Source: BMC Cancer. 2023 Jan 7;23:30. doi: 10.1186/s12885-022-10349-1 (PMC9826569; doi:10.1186/s12885-022-10349-1)
Supplement: Supplementary file 1 — Additional file 1. [file 12885_2022_10349_MOESM1_ESM.docx]

**Supplementary data**

Table e1: Disease status and diagnosis by decade

|  | | **Decade** | | | |  |  |  |  |  |  |  |
| --- | --- | --- | --- | --- | --- | --- | --- | --- | --- | --- | --- | --- |
| **Characteristic** | | **1980s (n=28)** | **1990s (n=52)** | **2000s (n=192)** | **2010s (n=261)** | **Total (N=533)** |  |  |  |  |  |  |
| **Primary Site** | | | | | | |  |  |  |  |  |  |
|  | Head/Neck | 18 (64%) | 32 (62%) | 96 (50%) | 121 (46%) | 267 (50%) |  |  |  |  |  |  |
|  | Left Lower Limb | 3 (11%) | 6 (10%) | 26 (14%) | 20 (8%) | 54 (10%) |  |  |  |  |  |  |
|  | Left Upper Limb | 2 (7%) | 3 (6%) | 14 (7%) | 26 (10%) | 45 (8%) |  |  |  |  |  |  |
|  | Right Lower Limb | 1 (4%) | 2 (4%) | 20 (10%) | 17 (7%) | 40 (8%) |  |  |  |  |  |  |
|  | Right Upper Limb | 2 (7%) | 4 (8%) | 8 (4%) | 18 (7%) | 32 (6%) |  |  |  |  |  |  |
|  | Trunk | 0 (0%) | 2 (4%) | 7 (4%) | 15 (6%) | 24 (5%) |  |  |  |  |  |  |
|  | Unknown | 2 (7%) | 3 (6%) | 21 (11%) | 44 (17%) | 70 (13%) |  |  |  |  |  |  |
|  | Unknown | 2 (7%) | 3 (6%) | 21 (11%) | 44 (17%) | 70 (13%) |  |  |  |  |  |  |
| **Stage** | | | | | | | 1 | 13 (50%) | 21 (44%) | 84 (44%) | 107 (41%) | 225 (43%) |
|  | 2A | 4 (15%) | 9 (19%) | 20 (11%) | 39 (15%) | 72 (14%) |  |  |  |  |  |  |
|  | 3 | 6 (23%) | 11 (23%) | 28 (15%) | 14 (5%) | 59 (11%) |  |  |  |  |  |  |
|  | 3A | 1 (4%) | 3 (6%) | 21 (11%) | 51 (20%) | 76 (15%) |  |  |  |  |  |  |
|  | 3B | 2 (8%) | 3 (6%) | 32 (17%) | 43 (16%) | 80 (15%) |  |  |  |  |  |  |
|  | 4 | 0 (0%) | 1 (2%) | 4 (2%) | 7 (3%) | 12 (2%) |  |  |  |  |  |  |
|  | Missing | 2 | 4 | 3 | 0 | 9 |  |  |  |  |  |  |
|  | Missing | 2 | 4 | 3 | 0 | 9 |  |  |  |  |  |  |
| **T stage** | | | | | | | T0 | 2 (7%) | 3 (6%) | 21 (11%) | 45 (17%) | 71 (13%) |
|  | T1 | 19 (68%) | 30 (58%) | 120 (62%) | 148 (57%) | 317 (59%) |  |  |  |  |  |  |
|  | T2 | 4 (14%) | 14 (27%) | 40 (21%) | 65 (25%) | 123 (23%) |  |  |  |  |  |  |
|  | T3 | 0 (0%) | 0 (0%) | 5 (3%) | 3 (1%) | 8 (2%) |  |  |  |  |  |  |
|  | Tx | 3 (11%) | 5 (10%) | 6 (3%) | 0 (0%) | 14 (3%) |  |  |  |  |  |  |
|  | Tx | 3 (11%) | 5 (10%) | 6 (3%) | 0 (0%) | 14 (3%) |  |  |  |  |  |  |
| **N stage** | | | | | | | N0 | 19 (68%) | 34 (65%) | 103 (54%) | 146 (56%) | 302 (57%) |
|  | N1 | 8 (29%) | 15 (29%) | 44 (23%) | 19 (7%) | 86 (16%) |  |  |  |  |  |  |
|  | N1a | 0 (0%) | 0 (0%) | 3 (2%) | 10 (4%) | 13 (2%) |  |  |  |  |  |  |
|  | N1b | 1 (4%) | 3 (6%) | 30 (16%) | 69 (26%) | 103 (19%) |  |  |  |  |  |  |
|  | N2 | 0 (0%) | 0 (0%) | 12 (6%) | 13 (5%) | 25 (5%) |  |  |  |  |  |  |
|  | N3 | 0 (0%) | 0 (0%) | 0 (0%) | 4 (2%) | 4 (1%) |  |  |  |  |  |  |
|  | N3 | 0 (0%) | 0 (0%) | 0 (0%) | 4 (2%) | 4 (1%) |  |  |  |  |  |  |
| **M stage** | | | | | | | M0 | 28 (100%) | 51 (98%) | 188 (98%) | 254 (97%) | 521 (98%) |
|  | M1 | 0 (0%) | 1 (2%) | 4 (2%) | 7 (3%) | 12 (2%) |  |  |  |  |  |  |
|  | M1 | 0 (0%) | 1 (2%) | 4 (2%) | 7 (3%) | 12 (2%) |  |  |  |  |  |  |
| **Mode of diagnosis** | | | | | | | Core | 0 (0%) | 0 (0%) | 9 (5%) | 23 (9%) | 32 (6%) |
|  | Curettage | 0 (0%) | 0 (0%) | 1 (1%) | 2 (1%) | 3 (1%) |  |  |  |  |  |  |
|  | Excision | 0 (0%) | 1 (2%) | 1 (1%) | 2 (1%) | 4 (1%) |  |  |  |  |  |  |
|  | Excision Complete | 19 (70%) | 30 (59%) | 84 (45%) | 86 (33%) | 219 (42%) |  |  |  |  |  |  |
|  | Excision Incomplete | 4 (15%) | 14 (27%) | 55 (30%) | 88 (34%) | 161 (31%) |  |  |  |  |  |  |
|  | Fna | 0 (0%) | 0 (0%) | 1 (1%) | 8 (3%) | 9 (2%) |  |  |  |  |  |  |
|  | Incision | 3 (11%) | 3 (6%) | 11 (6%) | 8 (3%) | 25 (5%) |  |  |  |  |  |  |
|  | Punch Biospy | 1 (4%) | 2 (4%) | 15 (8%) | 27 (10%) | 45 (9%) |  |  |  |  |  |  |
|  | Shave Biopsy | 0 (0%) | 0 (0%) | 5 (3%) | 12 (5%) | 17 (3%) |  |  |  |  |  |  |
|  | Other | 0 (0%) | 1 (2%) | 3 (2%) | 5 (2%) | 9 (2%) |  |  |  |  |  |  |
|  | Missing | 1 | 1 | 7 | 0 | 9 |  |  |  |  |  |  |
|  | Missing | 1 | 1 | 7 | 0 | 9 |  |  |  |  |  |  |
| **CXR staged** | | | | | | | No | 12 (100%) | 20 (61%) | 153 (83%) | 224 (99%) | 409 (90%) |
|  | Yes | 0 (0%) | 13 (39%) | 32 (17%) | 2 (1%) | 47 (10%) |  |  |  |  |  |  |
|  | Missing | 16 | 19 | 7 | 35 | 77 |  |  |  |  |  |  |
|  | Missing | 16 | 19 | 7 | 35 | 77 |  |  |  |  |  |  |
| **CT staged** | | | | | | | No | 13 (100%) | 18 (55%) | 66 (35%) | 97 (40%) | 194 (40%) |
|  | Yes | 0 (0%) | 15 (45%) | 123 (65%) | 148 (60%) | 286 (60%) |  |  |  |  |  |  |
|  | Missing | 15 | 19 | 3 | 16 | 53 |  |  |  |  |  |  |
|  | Missing | 15 | 19 | 3 | 16 | 53 |  |  |  |  |  |  |
| **PET staged** | | | | | | | No | 28 (100%) | 48 (92%) | 88 (47%) | 54 (22%) | 218 (42%) |
|  | Yes | 0 (0%) | 4 (8%) | 100 (53%) | 196 (78%) | 300 (58%) |  |  |  |  |  |  |
|  | Missing | 0 | 0 | 4 | 11 | 15 |  |  |  |  |  |  |
|  | Missing | 0 | 0 | 4 | 11 | 15 |  |  |  |  |  |  |
| **Lymphoscint** | | | | | | | No | 28 (100%) | 51 (98%) | 173 (90%) | 196 (75%) | 448 (84%) |
|  | Yes | 0 (0%) | 1 (2%) | 19 (10%) | 65 (25%) | 85 (16%) |  |  |  |  |  |  |
|  | Yes | 0 (0%) | 1 (2%) | 19 (10%) | 65 (25%) | 85 (16%) |  |  |  |  |  |  |
| **Sentinel node biopsy** | | | | | | | No | 28 (100%) | 52 (100%) | 173 (90%) | 209 (81%) | 462 (87%) |
|  | Yes | 0 (0%) | 0 (0%) | 19 (10%) | 49 (19%) | 68 (13%) |  |  |  |  |  |  |
|  | Missing | 0 | 0 | 0 | 3 | 3 |  |  |  |  |  |  |
|  | Missing | 0 | 0 | 0 | 3 | 3 |  |  |  |  |  |  |
| **Number of sentinel nodes** | | | | | | | 1 | NA | NA | 7 (41%) | 34 (65%) | 41 (59%) |
|  | 2 | NA | NA | 5 (29%) | 10 (19%) | 15 (22%) |  |  |  |  |  |  |
|  | 3 | NA | NA | 3 (18%) | 6 (12%) | 9 (13%) |  |  |  |  |  |  |
|  | 4 | NA | NA | 2 (12%) | 2 (4%) | 4 (6%) |  |  |  |  |  |  |
|  | Missing | 28 | 52 | 175 | 209 | 464 |  |  |  |  |  |  |
|  | Missing | 28 | 52 | 175 | 209 | 464 |  |  |  |  |  |  |
| **Sentinel node status** | | | | | | | Negative | 0 (0%) | 0 (0%) | 13 (72%) | 34 (71%) | 47 (71%) |
|  | Positive | 0 (0%) | 0 (0%) | 5 (28%) | 14 (29%) | 19 (29%) |  |  |  |  |  |  |
|  | Missing | 28 | 52 | 174 | 213 | 467 |  |  |  |  |  |  |
|  | Missing | 28 | 52 | 174 | 213 | 467 |  |  |  |  |  |  |
| **In-transit/satellite disease** | | | | | | | No | 28 (100%) | 51 (98%) | 171 (90%) | 229 (94%) | 479 (93%) |
|  | Yes | 0 (0%) | 1 (2%) | 18 (10%) | 15 (6%) | 34 (7%) |  |  |  |  |  |  |
|  | Missing | 0 | 0 | 3 | 17 | 20 |  |  |  |  |  |  |
|  | Missing | 0 | 0 | 3 | 17 | 20 |  |  |  |  |  |  |
| **Follow up imaging** | | | | | | | No | 0 (0%) | 2 (11%) | 39 (27%) | 36 (17%) | 77 (21%) |
|  | Yes | 0 (0%) | 16 (89%) | 103 (73%) | 171 (83%) | 290 (79%) |  |  |  |  |  |  |
|  | Missing | 28 | 34 | 50 | 54 | 166 |  |  |  |  |  |  |
|  |  |  |  |  |  |  |  |  |  |  |  |  |

Table e2: Cumulative Incidence estimates (%) with 95% confidence intervals, regional and distant recurrence.

| **Years** | **Local recurrence** | **Loco-regional recurrence** | **Distant recurrence** |
| --- | --- | --- | --- |
| 1 | 8 (6-10) | 24 (20-27) | 16 (13-19) |
| 2 | 10 (7-12) | 31 (27-35) | 25 (21-29) |
| 3 | 10 (8-13) | 32 (28-36) | 28 (24-32) |
| 4 | 10 (8-13) | 32 (28-36) | 28 (24-33) |
| 5 | 10 (8-13) | 32 (28-36) | 30 (26-34) |
| 10 | 11 (8-14) | 33 (29-37) | 1. 26-35) |

Figure e1 Overall Survival and disease free survival by cancer with unknown primary (CUP)

| 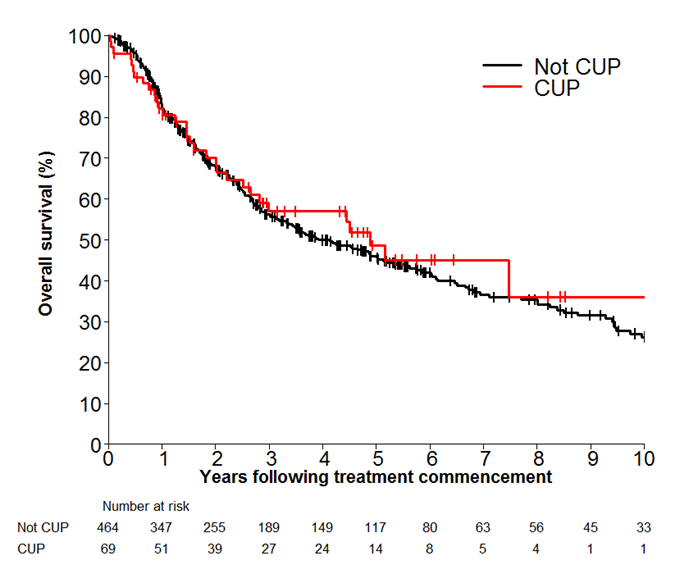 | 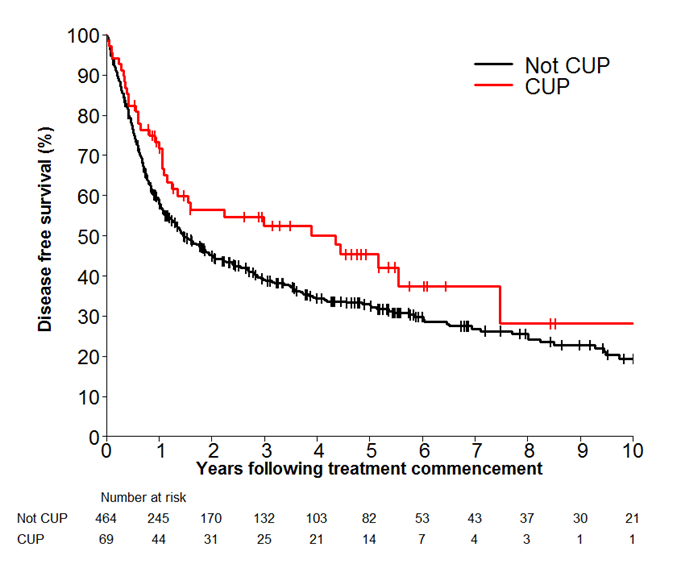 |
| --- | --- |

Figure e2: Disease Free Survival and Over Survival by decade


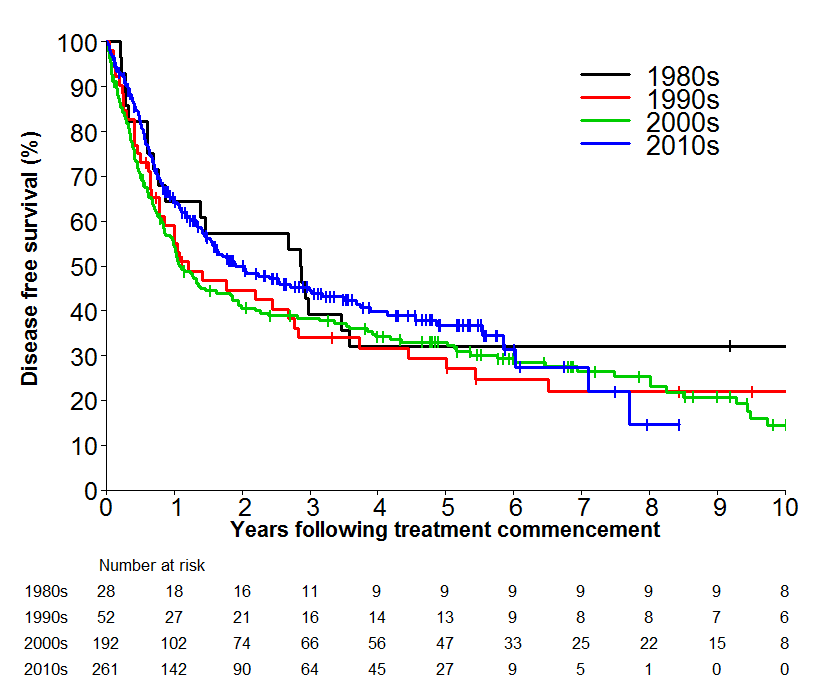

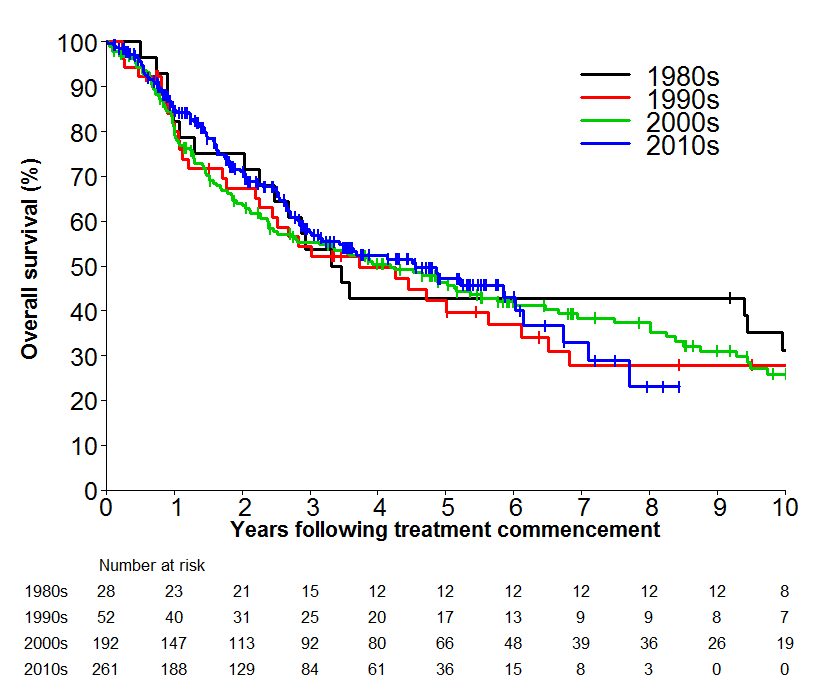


Table e3: Cumulative incidence estimates (%) with 95% confidence interval for local recurrence by stage

| **Years** | **Local recurrence** | | | | **Death** | | | |
| --- | --- | --- | --- | --- | --- | --- | --- | --- |
|  | **Stage I** | **Stage II** | **Stage III** | **Stage IV** | **Stage I** | **Stage II** | **Stage III** | **Stage IV** |
| 1 | 8 (5-12) | 7 (3-15) | 22 (16-27) | 45 (15-72) | 6 (4-10) | 21 (12-31) | 5 (2-8) | 0 (0-0) |
| 2 | 18 (13-24) | 18 (9-28) | 40 (33-47) | 72 (15-95) | 8 (5-12) | 26 (16-37) | 6 (3-9) | 9 (0-37) |
| 3 | 27 (21-33) | 23 (13-35) | 52 (45-59) | NA (NA-NA) | 9 (5-13) | 26 (16-37) | 7 (4-11) | NE |
| 4 | 34 (27-41) | 32 (20-45) | 54 (47-61) | NA (NA-NA) | 9 (5-13) | 26 (16-37) | 7 (4-11) | NE |
| 5 | 38 (31-45) | 35 (22-48) | 58 (50-65) | NA (NA-NA) | 9 (5-13) | 26 (16-37) | 7 (4-11) | NE |
| 10 | 59 (49-67) | 47 (26-65) | 75 (64-84) | NA (NA-NA) | 9 (5-13) | 29 (18-42) | 7 (4-11) | NE |

Table e4: OS estimates (%) by decade of initial treatment

| **Year** | **1980s** | **1990s** | **2000s** | **2010s** |
| --- | --- | --- | --- | --- |
| 1 | 82 (62 -92) | 82 (68 -90) | 80 (74 -85) | 85 (80 -89) |
| 2 | 75 (55 -87) | 67 (52 -79) | 64 (57 -71) | 71 (64 -77) |
| 3 | 54 (34 -70) | 54 (39 -67) | 55 (48 -62) | 58 (50 -64) |
| 4 | 43 (25 -60) | 50 (35 -63) | 50 (43 -58) | 52 (45 -59) |
| 5 | 43 (25 -60) | 42 (28 -56) | 46 (39 -54) | 47 (39 -55) |
| Median (Years) | 3.4 (2.3 - 9.9) | 3.7 (2.2 - 6.1) | 4.2 (2.5 - 5.8) | 4.6 (3.0 - 6.1) |

Table e5: DFS estimates (%) by decade of initial treatment

| **Year** | **1980s** | **1990s** | **2000s** | **2010s** |
| --- | --- | --- | --- | --- |
| 1 | 64 (44 -79) | 55 (40 -67) | 55 (47 -61) | 64 (58 -70) |
| 2 | 57 (37 -73) | 45 (31 -58) | 41 (34 -48) | 50 (43 -56) |
| 3 | 39 (22 -57) | 34 (21 -47) | 38 (31 -45) | 45 (38 -51) |
| 4 | 32 (16 -49) | 32 (19 -45) | 34 (27 -41) | 40 (33 -47) |
| 5 | 32 (16 -49) | 29 (17 -43) | 33 (26 -40) | 37 (29 -44) |
| Median (Years) | 2.9 (0.8 - 3.6) | 1.2 (0.8 - 2.8) | 1.1 (0.9 - 1.9) | 1.9 (1.5 - 3.2) |
